# Improving exercise motivation and physical fitness in college students through a long-term mindfulness-enhanced Tai Chi Chuan program: a randomized controlled trial

**DOI:** 10.7717/peerj.20602

**Published:** 2026-01-09

**Authors:** Ping Qu, Xiaoqing Zhu, Ting Zhu, Hui Zhou, Minghua Huang, Feng Pan, Xiaoyan Wang, Jingsi Wen, Yang Liu, Yu Zhang, Fangbin Li, Yuyin Wang

**Affiliations:** 1 Department of Physical Education, Sun Yat-Sen University, Guangzhou, China; 2 Department of Psychology, Sun Yat-Sen University, Guangzhou, China; 3 Department of Physical Education, Shenzhen Polytechnic University, Shenzhen, China; 4 Department of Sports Science and Physical Education, Chinese University of Hong Kong, Hong Kong, China

**Keywords:** Tai Chi Chuan, Mindfulness, Exercise motivation, Physical fitness

## Abstract

**Background:**

While Tai Chi Chuan (TCC) is widely recognized for its physical and mental health benefits, its mindful components are often overlooked in traditional training. Mindfulness-enhanced Tai Chi Chuan (MTCC) has been proposed to address this limitation, yet little research has explored its long-term impact on exercise motivation and physical fitness. This study extends previous findings by investigating the sustained effects of a 24-week MTCC intervention in improving physical and mental health outcomes among college students, with a focus on long-term sustainability and motivation maintenance that distinguishes it from our prior short-term research.

**Methods:**

A randomized controlled trial was conducted with 80 college students assigned to either an MTCC group or a traditional TCC group. Seventy-one participants completed the allocated intervention, while nine participants dropped out for personal reasons. No serious adverse events (SAEs) were reported. The MTCC program was delivered in two stages over 24 weekly sessions. The first stage introduced participants to mindfulness-enhanced Taiyi Mirrored-heart Chuan, emphasizing foundational postures and mindfulness principles. The second stage involved traditional TCC training to consolidate the skills and motivation gained during the first stage. Outcome measures, including exercise motivation, physical fitness, mindfulness and subjective well-being, were assessed pre- and post-intervention.

**Results:**

Repeated measures analysis of variance (ANOVA) indicated that participants in the MTCC group demonstrated significantly greater improvements in all measured outcomes compared to the traditional TCC group. Specifically, the 24-week intervention showed larger effect sizes in physical fitness (*η*^2^_*p*_ = 0.224) compared to our prior study (*η*^2^_*p*_ = 0.033–0.210), highlighting the value of long-term intervention.

**Conclusion:**

These findings highlight the long-term benefits of integrating mindfulness into TCC practice, particularly in fostering intrinsic motivation for physical activity and enhancing overall well-being. The study underscores the potential of MTCC as a sustainable intervention for promoting holistic health in college students and its applicability in educational and wellness settings.

**Clinical review registration:**

https://www.chictr.org.cn/, identifier ChiCTR2200058449.

## Introduction

Physical and mental health challenges among college students, stemming from academic pressures, lifestyle factors, and transitional uncertainties, represent a significant global public health concern (Tosevski, Milovancevic & Gajic, 2010; Deasy et al., 2014; Badger, Quatromoni & Morrell, 2019; Yang et al., 2022). Mind-body exercises like Tai Chi Chuan (TCC) offer a promising approach to address these multifaceted issues, demonstrating benefits for both physical fitness and psychological well-being in this population (Li et al., 2023; Caldwell et al., 2011). However, evidence from randomized controlled trial (RCT) has also shown that traditional TCC only improved physical indicators (flexibility and balance) among college students, with no significant effects on psychological symptoms or mindfulness (Zheng et al., 2015). Moreover, a meta-analysis of 23 RCTs revealed that TCC outperforms non-mindful exercise in reducing anxiety and depression only when explicit mindfulness elements (*e.g.*, intentional breathing, present-moment awareness) are integrated (Yin et al., 2023). This evidence consistently emphasizes that the mindful essence intrinsic to TCC practice is frequently underemphasized in conventional training paradigms, limiting its full therapeutic potential, particularly for mental health outcomes.

Mindfulness-based Tai Chi Chuan (MTCC) has emerged as an optimized intervention that embeds mindfulness principles into traditional TCC practice. Preliminary evidence from RCTs has demonstrated MTCC’s capacity to reduce psychological distress and enhance physical performance in adolescents and older adults, with intervention durations ranging from 8 weeks to 6 months (Zhang et al., 2018; Jiayuan et al., 2022; Zhang et al., 2022). In our previous study, we demonstrated that a 10-week mindfulness-enhanced Tai Chi Chuan program led to significantly improvements in physical fitness and mental well-being among college students compared to traditional TCC (Qu et al., 2024). Importantly, research suggests that the effectiveness of mindfulness-based interventions in improving mental health is closely related to the length of the intervention (Fu et al., 2024; Wang et al., 2023a; Wang et al., 2023b). Studies on TCC have also shown that medium- to long-term practice durations are more effective in promoting physical and cognitive functions (Nan et al., 2024; Walsh et al., 2015). These findings indicate that longer programs provide participants with additional opportunities to internalize mindfulness principles and establish a consistent practice routine, ultimately resulting in more substantial and enduring benefits.

With these insights in mind, we developed a long-term MTCC program to maximize its impact on both physical and mental health. Longer duration allows for deeper integration of mindfulness and physical practice, facilitating more substantial improvements in well-being. Additionally, prolonged mindfulness practice enhances self-awareness, emotional regulation, and resilience, which are critical for managing the stressors faced by college students (Bai et al., 2020; Ramasubramanian, 2016). However, maintaining participation in long-term programs can be challenging due to factors such as exercise monotony and low motivation (Du, Roberts & Liu, 2023). Given that low physical activity is common among college students, boosting exercise motivation is essential for increasing participation, which, in turn, improves mental health and life satisfaction (Li et al., 2024; Liu, Zhu & Jiang, 2021). Mindfulness-based interventions have been shown to enhance motivation and engagement (Lynn et al., 2022). Therefore, our program specifically focused on utilizing mindfulness to increase exercise motivation and sustain long-term engagement, aiming to provide a more engaging experience that fosters consistent participation and maximizes health benefits.

In this randomized controlled trial (RCT), we aimed to investigate the effects of the long-term MTCC intervention program on subjective well-being, mindfulness, exercise motivation, and physical fitness among college students. This program was designed in two stages to accommodate both initial learning and skill consolidation. Although the study’s current implementation involved a 24-week program, this structure was chosen based on practical considerations (specifically, the school teaching schedule in this study). The program itself remains adaptable, and future implementations may vary in duration or structure, emphasizing its practical application within real-world settings. We hypothesize that this long-term MTCC program will result in greater improvements in physical and mental health outcomes compared to traditional TCC interventions.

## Materials & Methods

### Participants

Sample size calculation was performed using G*Power 3.1.9.2 (Faul et al., 2007) for a 2 (between-subjects factors: MTCC group *vs.* TCC group) ×2 (within-subjects factors: pre-test *vs.* post-test) repeated-measures analysis of variance (ANOVA). Assuming a medium effect size of *f* = 0.25, a power of 0.90 at a significance level of *α* = 0.05, and a correlation among repeated measures of *r* = 0.5, a minimum sample size of 46 was needed to provide adequate power for the analyses.

The inclusion criteria were: (1) first and second-year university students; (2) age ≥ 18 years; and (3) willingness to participate in this study. The exclusion criteria included current and previous diagnosis of (1) chronic physical conditions (*e.g.*, cardiovascular disease, hypertension, diabetes); (2) major psychiatric conditions (*e.g.*, schizophrenia, bipolar disorders); (3) physical disability; and (4) engagement in regular Tai Chi Chuan training or other high-intensity exercise (defined as practicing at least twice a week for a minimum of 30 min per session).

**Figure 1:**
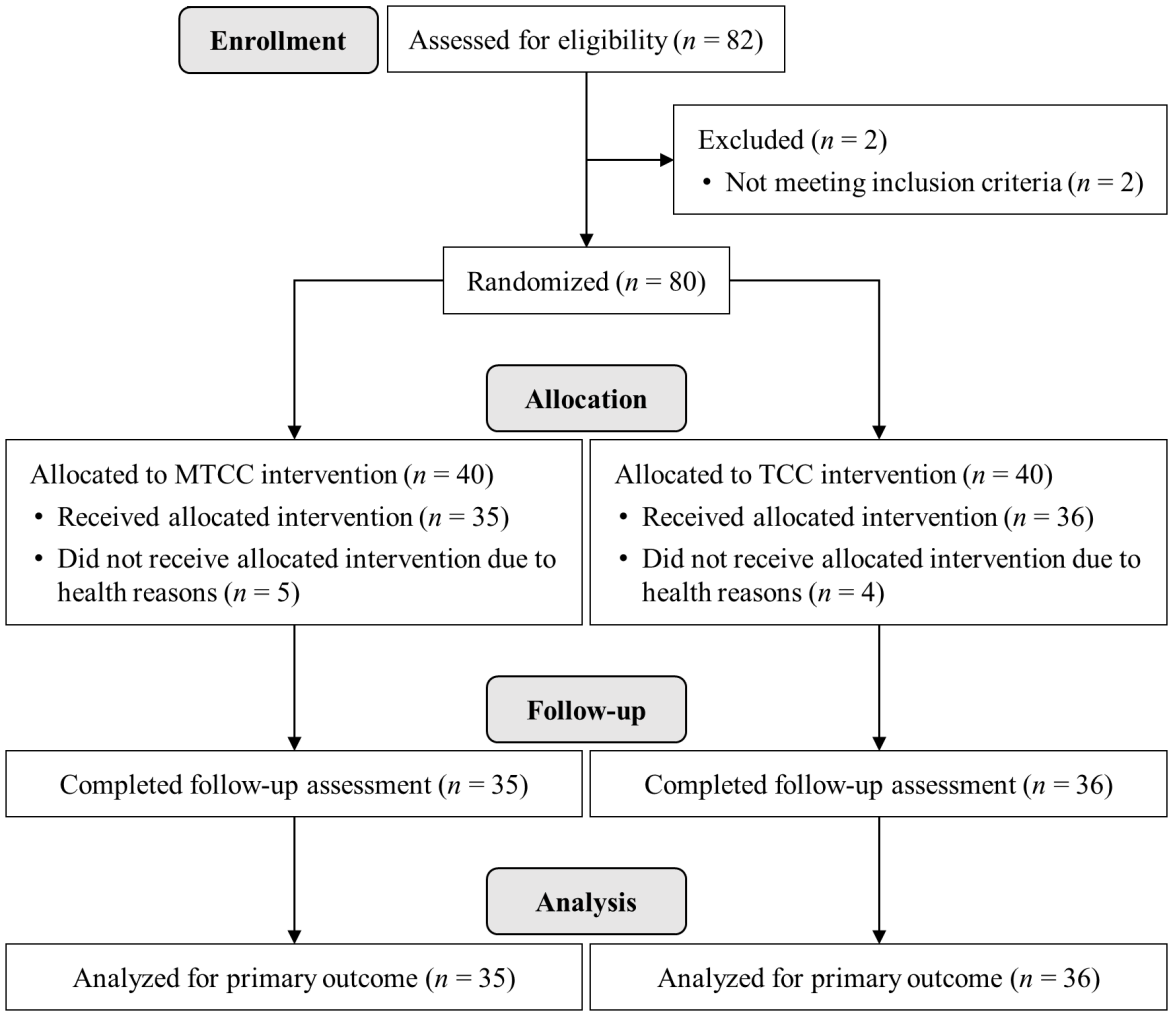
CONSORT flowchart.

As illustrated in Fig. 1, a total of 80 participants who met the criteria were randomly assigned to MTCC group or TCC group in a 1:1 ratio, stratified by age and gender. Randomization was carried out using the random number generator in SAS. Seventy-one participants completed the allocated intervention, while nine participants (five in MTCC group and four in TCC group) dropped out for personal reasons and were removed from the analyses. No significant differences were found between completers and dropouts at pre-test (*ps* > 0.05). The final valid sample consisted of 35 participants in the MTCC group (12 males and 23 females; mean age = 18.17, *SD* = 0.66) and 36 participants in the TCC group (eight males and 28 females; mean age = 18.08, *SD* = 0.65).

### Interventions

The MTCC intervention program was established by a professional team consisting of physical education and sports specialists, psychologists, and martial arts instructors. This program comprised two interconnected stages, with a total of 24 weekly sessions, each lasting 90 minutes—representing a key advancement from our prior 10-week single-stage intervention (Qu et al., 2024).

The beginner stage was conducted over 9 weeks during the fall semester, involving training in the 16-form Taiyi Mirrored-heart Chuan, adapted from Yang-style Tai Chi Chuan, Yijinjing and Tai Chi Tuishou. This stage emphasized mindfulness principles (*e.g.*, breath awareness, non-judgmental movement observation) to build foundational body awareness, making it suitable for both beginners and those with limited exercise experience.

The consolidation stage was conducted over 15 weeks during the spring semester, involving training in the 24-form Yang-style Tai Chi Chuan. This stage aimed to consolidate mindfulness skills into traditional routines. Notably, both groups practiced the same 24-form TCC in this stage, demonstrating that mindfulness principles established in the first stage can be effectively integrated into conventional TCC without reliance on specialized forms.

The details of the MTCC program were as follows: The first session provided theoretical education about TCC and mindfulness. Each subsequent session involved four main contents: (1) a 25-min preparation, including mindful sharing and warm-up exercise; (2) a 20-min training content review; (3) a 35-min formal training, including TCC training and mindfulness practice (*e.g.*, breathing space, loving-kindness meditation); and (4) a 10-min ending part including relaxation exercise and a course summary. Participants were also encouraged to engage in self-directed TCC training and mindfulness practice between sessions, with supplementary reading materials provided to deepen understanding. More details about the MTCC program can be found in the Supplementary Material.

The traditional TCC intervention program shared the same duration and structure as the MTCC program. The TCC trainings consisted of the 16-form Yang-style Tai Chi Chuan in the beginner stage and the 24-form Yang-style Tai Chi Chuan in the consolidation stage. The main distinction was the absence of Taiyi Mirrored-heart Chuan and structured mindfulness practices (*e.g.*, mindful sharing, breathing space) in the TCC program. More details about the TCC program can be found in the Supplementary Material.

### Procedure

To evaluate the effectiveness of the long-term MTCC intervention, a randomized controlled trial with repeated measures was conducted. All participants provided written informed consent to participate in the study. After the pre-test (T1), participants were assigned to either the MTCC group or the TCC group using stratified randomization. Participants in both groups received a 24-week intervention led by qualified university physical education instructors and subsequently completed the post-test (T2). Pre- and post-test measurements included assessments of subjective well-being, mindfulness, exercise motivation and physical fitness. Approval for the study was obtained from the Ethical Committee of the Department of Psychology at Sun Yat-Sen University (Approved No. 2021-1105-0213).

### Measures

#### Exercise motivation

The 15-item Simplified Version of the Motives for Physical Activities Measure-Revised (MPAM-R; Chen et al., 2013, revived from Ryan et al., 1997) was used to assess participants’ motivations for exercise adherence. This scale measures five distinct motives: fitness, competence, enjoyment, appearance, and social. Participants rated their responses on a 5-point Likert scale from 1 (not at all true for me) to 5 (very true for me). A total score was computed by summing the scores of the individual items, with higher scores indicating a higher level of general exercise motivation. The Cronbach’s alpha in this study was 0.892 (T1) and 0.862 (T2).

#### Physical fitness

Physical fitness assessment including body fat percentage, vital capacity, 50 m sprint, sit and reach, standing long jump, 1 min sit-ups, and endurance running (800 m/1000 m). The results recorded at each assessment were first converted into a *Z*-score based on the mean and standard deviation, which were then converted into a *T*-score with a mean of 50 and a standard deviation of 10. *T*-scores of body fat percentage and endurance running were further reversed (*i.e.,* 100 minus *T*-score) to obtain positive indicators. A total score of general physical fitness was computed by summing the *T*-scores or reversed *T*-scores of each assessment, with higher scores indicating greater physical fitness.

#### Mindfulness

The 20-item Chinese version of Five Facet Mindfulness Questionnaire-Short Form (FFMQ-SF; Hou et al., 2014) was used to assess participants’ general tendency to be mindful in daily life. This questionnaire measures five facets of mindfulness: observing (*e.g.*, “I notice the smells and aromas of things”), describing (*e.g.*, “I’m good at finding words to describe my feelings”), acting with awareness (*e.g.*, “I am easily distracted”), nonjudging of inner experience (*e.g.*, “I tell myself I shouldn’t be feeling the way I’m feeling”), and nonreactivity to inner experience (*e.g.*, “In difficult situations, I can pause without immediately reacting”). Participants rated their responses on a 5-point Likert scale from 1 (never or very rarely true) to 5 (very often or always true). A total score was computed by summing the scores of the individual items, with higher scores indicating a greater level of mindfulness. The Cronbach’s alpha in this study was 0.732 (T1) and 0.809 (T2).

#### Subjective well-being

The 9-item Index of Well-Being (IWB; Campbell, Converse & Rodgers, 1976) was used to assess the degree of subjective well-being. This scale consists of two parts: (1) the index of general affect, measured by eight semantic–differential items (*e.g.*, boring *vs.* interesting); and (2) the index of life satisfaction, measured by a single item (*i.e.,* “How satisfied or dissatisfied are you with your life as a whole?”). Participants rated their responses on a scale from 1 (*e.g.*, boring/dissatisfied) to 7 (*e.g.*, interesting/satisfied). A composite index of well-being was computed by summing the average scores of the index of general affect (weight = 1) and the index of life satisfaction (weight = 1.1), with scores ranging from 2.1 (extremely unhappy) to 14.7 (extremely happy). The Cronbach’s alpha in this study was 0.885 (T1) and 0.931 (T2).

### Data analyses

First, we conducted independent *t*-tests and chi-square tests to assess baseline differences between the MTCC group and the TCC group. Second, we conducted a 2 (group: MTCC *vs.* TCC) ×2 (time: pre-test *vs.* post-test) repeated-measures ANOVA to compare the intervention effects of the two groups on the outcome measurements. When the time × group interaction effect was significant, simple effect analyses were conducted to determine the statistical significance of both intergroup differences and intragroup differences across time points. Effect sizes were reported by means of partial eta squared (*η*^2^_*p*_), with the criteria for effect magnitude defined as follows: small (0.01), moderate (0.06), large (0.14), in accordance with Cohen (1988). All statistical analyses were conducted using IBM SPSS 27.

## Results

### Descriptive statistics

Table 1 presents the descriptive statistics for all variables in the two groups. No significant baseline differences were observed between the MTCC group and the TCC group in age, sex or other study variables (*ps* > 0.05). To address potential ceiling effects (baseline MPAM-R mean = 62/75) and eliminate regression artifacts, we tested baseline and group interaction effects for all outcomes. No significant baseline and group interactions were observed (*ps* > 0.05).

**Table 1:** Results of descriptive statistics.

	MTCC (*n* = 35)	TCC (*n* = 36)	*t*/*χ*^2^	*p*
Age	18.17 ± 0.66	18.08 ± 0.65	−0.565	0.574
Gender, *n* (%)			1.276	0.259
Male	12 (34.3%)	8 (22.2%)		
Female	23 (65.7%)	28 (77.8%)		
Subjective well-being	10.45 ± 1.86	11.18 ± 1.56	1.788	0.078
Mindfulness	68.83 ± 9.01	66.61 ± 7.99	−1.098	0.276
Exercise motivation	61.94 ± 8.52	61.78 ± 9.16	−0.079	0.938
Physical fitness	334.94 ± 42.87	328.78 ± 35.02	−0.664	0.509

### Intervention effect

**Figure 2:**
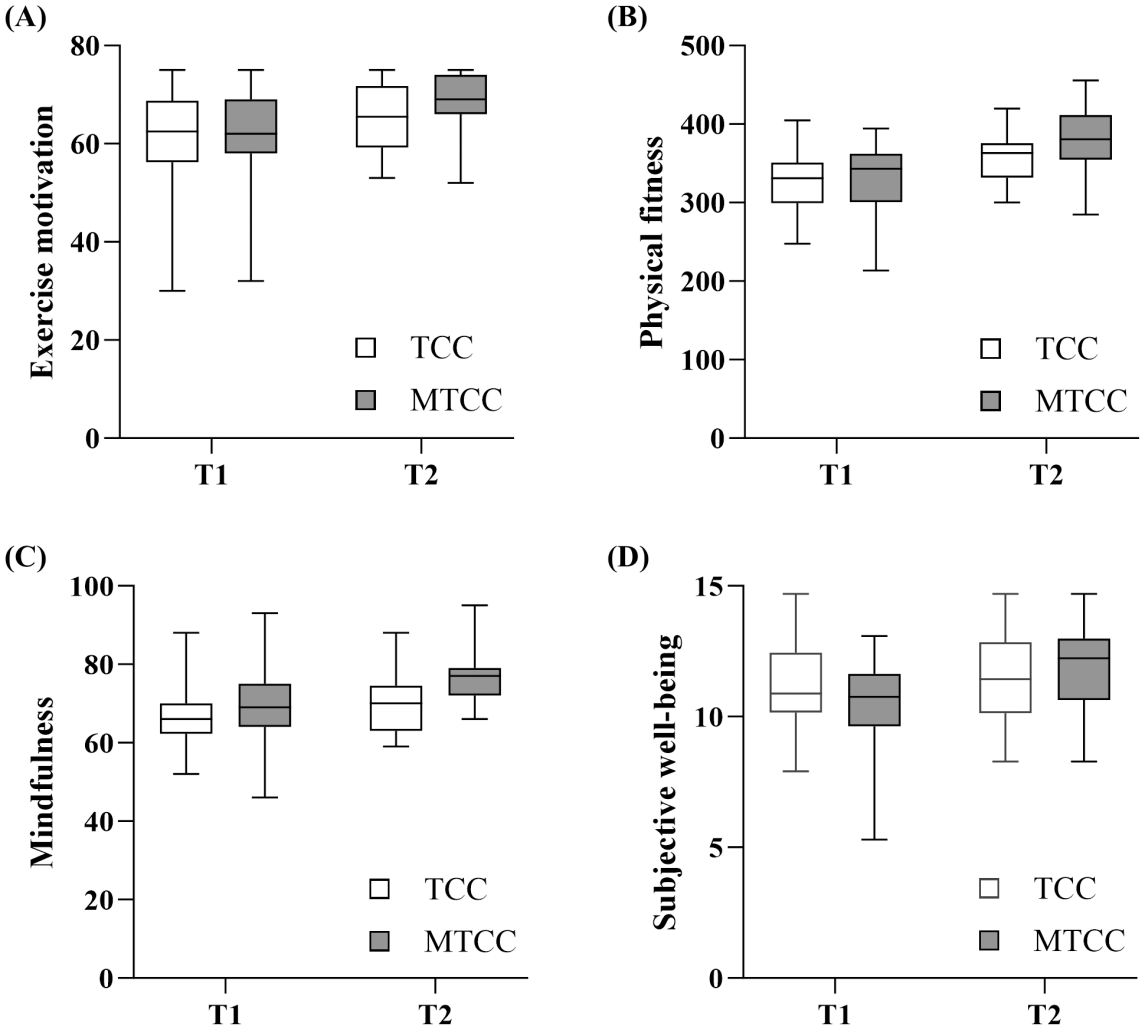
(A–C) Changes in all variables across time among the groups.

Repeated measures ANOVA indicated significant interactions between time and group in exercise motivation (*F* (1, 69) = 5.736, *p* = 0.019, *η*^2^_*p*_ = 0.077), physical fitness (*F* (1, 69) = 19.900, *p* < 0.001, *η*^2^_*p*_ = 0.224), mindfulness (*F* (1, 69) = 6.494, *p* = 0.013, *η*^2^_*p*_ = 0.086), and subjective well-being (*F* (1, 69) = 9.572, *p* = 0.003, *η*^2^_*p*_ = 0.122). Simple effect analyses revealed that for the MTCC group, the level of subjective well-being increased significantly after the intervention (*F* (1, 69) = 24.771, *p* < 0.001, *η*^2^_*p*_ = 0.264); however, there was no significant change in the TCC group (*p* > 0.05). Moreover, the level of mindfulness, exercise motivation, and physical fitness increased significantly in both groups. The details are shown in Table 2 and Fig. 2.

**Table 2:** Results of repeated-measures ANOVA.

	MTCC (*n* = 35)		TCC (*n* = 36)		*F*	*p*	${\eta }_{p}^{2}$
	Pretest	Posttest	Pretest	Protest			
Subjective well-being	10.45 ± 1.86	12.05 ± 1.69	11.18 ± 1.56	11.38 ± 1.81	9.572	0.003	0.122
Mindfulness	68.83 ± 9.01	76.77 ± 6.39	66.61 ± 7.99	69.81 ± 7.56	6.494	0.013	0.086
Exercise motivation	61.94 ± 8.52	69.17 ± 5.52	61.78 ± 9.16	65.03 ± 6.84	5.736	0.019	0.077
Physical fitness	334.94 ± 42.87	380.97 ± 39.51	328.76 ± 35.02	355.76 ± 29.94	19.899	<0.001	0.224

To assess the robustness of intervention effects against missing data (nine participants withdrew: five in the MTCC group, four in the TCC group), we conducted a sensitivity analysis using the Last Observation Carried Forward (LOCF) method—imputing missing post-test data with participants’ pre-test (T1) values for all outcomes. Repeated measures ANOVA on the LOCF-imputed dataset confirmed that the significant time and group interaction effects remained consistent with the original analysis of complete data (*ps* < 0.05), indicating the intervention effects were not substantially influenced by missing data.

## Discussion

This randomized controlled study aimed to examine the effects of a long-term mindfulness-enhanced Tai Chi Chuan (MTCC) intervention on exercise motivation, physical fitness, and subjective well-being among college students. Consistent with our hypotheses, the results demonstrated that participants who engaged in the MTCC program exhibited significant improvements across all measured outcomes compared to those practicing traditional TCC. These findings reinforce the notion that while TCC is well-recognized for its physical and mental health benefits, integrating structured mindfulness components can further amplify its effectiveness.

Unlike conventional TCC or short-term MTCC programs, our 24-week intervention uniquely scaffolds mindfulness acquisition: Initial Taiyi training primes body awareness, while subsequent traditional TCC practice embeds mindfulness into complex movements. This phased approach may explain the significant gains in intrinsic motivation (*e.g.*, MPAM-R ’enjoyment’ subscale), a predictor of long-term adherence. This design aligns with the principles of skill transfer (Issurin, 2013; Pierce, Gould & Camiré, 2016), where foundational mindfulness skills developed in simplified routines (Taiyi Mirrored-heart Chuan) are generalized to more complex traditional forms, enhancing sustainability.

Traditional TCC has often been viewed as a practice primarily suited for older adults due to its low-impact nature (Park et al., 2023). However, the promotion of TCC among young adults has faced challenges related to motivation and adherence (Caldwell et al., 2016; Converse et al., 2020). Our findings suggest that MTCC shows potential as a more effective alternative for younger populations by enhancing exercise motivation alongside physical fitness improvements. By incorporating mindfulness principles, MTCC fosters greater body awareness, which may facilitate engagement with physical activity and contribute to long-term exercise adherence. Given the prevalence of sedentary lifestyles among young adults (Zhang et al., 2023), MTCC offers a compelling intervention for addressing these concerns. Furthermore, the mindfulness components of MTCC appear to enhance self-regulation and perceived exercise self-efficacy, factors critical for sustaining long-term physical activity engagement (Neace et al., 2022; Smart et al., 2016).

Beyond physical fitness, our results also demonstrated that the MTCC program elicited greater improvements in subjective well-being and mindfulness compared to TCC alone. The significant interaction effects observed suggest that integrating mindfulness into TCC training enhances its psychological benefits. TCC is widely acknowledged for its positive effects on mental well-being (Chen, Zhang & Yu, 2022; Wang et al., 2023a; Wang et al., 2023b), and previous research has indicated that adding mindfulness practices can further optimize these benefits (Wang et al., 2016). A distinguishing feature of our MTCC program is the deliberate and structured incorporation of mindfulness elements, particularly through Taiyi Mirrored-heart Chuan, a technique developed by our research team to synchronize breathing, consciousness, and movement.

### Limitations and external validity

This trial possesses notable methodological strengths, including its randomized controlled design and ecologically valid implementation within a university curriculum—providing actionable insights for integrating mindfulness-based exercise in educational settings. However, three limitations merit discussion. First, while adequately powered for primary outcomes, recruitment restricted to college students may limit generalizability to populations with distinct lifestyles (*e.g.*, older adults or occupational cohorts). Second, the absence of interim and post-intervention follow-up assessments precluded analysis of temporal effect trajectories and long-term sustainability. The 12-month follow-up assessment planned in the trial protocol was not conducted due to high participant attrition. Nevertheless, the 24-week intervention duration substantially exceeds typical mind-body trials (*e.g.*, 8–12 weeks; Wang et al., 2016), offering novel evidence on maintained effects. Third, physical fitness assessments were administered by the same instructors who delivered the intervention, with assessors not blinded to group allocation. This may introduce positive assessment bias. Future studies should: (a) validate MTCC in diverse populations; (b) optimize follow-up strategies to enhance retention and capture long-term effects; and (c) employ assessors independent of intervention delivery and implement blinding procedures to mitigate assessment bias.

This study has several implications. First, the successful integration of mindfulness into TCC underscores the potential for applying similar enhancements to other physical activities to improve engagement and adherence. Second, the long-term and phased design of MTCC appears critical for its efficacy. Unlike shorter interventions, the extended duration allows participants to progressively deepen their practice, enhancing retention and health benefits. This structured, multi-stage approach may serve as a model for developing other sustained interventions aimed at fostering holistic health.

## Conclusions

This trial extends the empirical evidence of mindfulness-enhanced Tai Chi Chuan (MTCC). It confirms that a 24-week, two-stage intervention effectively improves college students’ exercise motivation, physical fitness, and subjective well-being. This fills a key gap in long-term adherence research, compared with our previous 10-week beginner-focused study (Qu et al., 2024), and highlights the value of integrating structured mindfulness into traditional Tai Chi for extended periods to boost holistic health. Practically, universities should pilot this 24-week, two-stage program as a physical education elective. Its delivery fits existing curriculum frameworks and can address young adults’ sedentary lifestyles by enhancing intrinsic exercise adherence. For future trials, regular booster sessions (*e.g.*, monthly 1-hour refresher classes post-intervention) and 6- to 12-month follow-ups are needed. These steps will verify if MTCC’s benefits last long-term, which is critical to confirming its scalability as a population-level health promotion tool.

##  Supplemental Information

10.7717/peerj.20602/supp-1 Supplemental Information 1 Intervention program

10.7717/peerj.20602/supp-2 Supplemental Information 2 Raw data The raw data contains pre- and post-test data for exercise motivation, physical fitness, mindfulness, and subjective well-being from 71 participants (35 in the MTCC group, 36 in the TCC group) included in the final analysis. These data were used for statistical analyses to compare the intervention effects between two groups on outcome measurements.

10.7717/peerj.20602/supp-3 Supplemental Information 3 Clinical trial registration

10.7717/peerj.20602/supp-4 Supplemental Information 4 CONSORT checklist

10.7717/peerj.20602/supp-5 Supplemental Information 5 Codebook

## Additional Information and Declarations

### Data Availability

The following information was supplied regarding data availability: The raw measurements are available in the Supplementary File.

### Clinical Trial Registration

The following information was supplied regarding Clinical Trial registration: ChiCTR2200058449.
