# Supplementary material for: Improving exercise motivation and physical fitness in college students through a long-term mindfulness-enhanced Tai Chi Chuan program: a randomized controlled trial
Source: PeerJ. 2026 Jan 9;14:e20602. doi: 10.7717/peerj.20602 (PMC12794632; doi:10.7717/peerj.20602)
Supplement: Supplemental Information 1 [file peerj-14-20602-s001.docx]

| Supplemental Material | | | |
| --- | --- | --- | --- |
|  |  | **MTCC program**  **(Beginner stage: Session 1 ‒ 9)** | **TCC program** |
| Session 1 | Major contents | 1. Introduction: Tai Chi Chuan and mindfulness 2. Mindfulness practice: mindful breathing | 1. Introduction: Tai Chi Chuan |
|  | Homework | 1. Reding materials:    - Introduction to Taiyi Mirror Heart Chuan    - Introduction to mindfulness 2. Mindfulness practice: mindful breathing 3. Tai Chi Chuan practice: Holding Fist | 1. Tai Chi Chuan practice: Holding Fist |
| Session 2 | Major contents | 1. Review & Discussion 2. Taiyi Mirror Heart Chuan: form 0−2    - Preparation: take three deep breaths    - Commencing Form (起势)    - Lifting and Standing (顶天立地式) 3. Mindfulness practice: awareness of body sensations | 1. 16-form Yang-style Tai Chi Chuan: form 0-2    - Preparation: take three deep breaths    - Commencing Form (起势)    - Part the Wild Horse's Mane on Both Side (左右野马分鬃) |
|  | Homework | 1. Reding materials:    - Challenges and coping strategies in Tai Chi Chuan practice    - The story of Yang Luchan, the founder of Yang-style Tai Chi Chuan 2. Taiyi Mirror Heart Chuan practice 3. Mindfulness practice: mindfulness activities in daily life | 1. 16-form Yang-style Tai Chi Chuan practice |
| Session 3 | Major contents | 1. Review & Discussion 2. Taiyi Mirror Heart Chuan: form 3−4    - Three Plates Falling on the Floor (三盘落地式)    - Grasp the Bird’s Tail – Left (左揽雀尾) 3. Mindfulness practice: body scan | 1. Review 2. 16-form Yang-style Tai Chi Chuan: form 3-4    - White Crane Spreads its Wings (白鹤亮翅)    - Brush Knee and Twist Step on Both Side (左右搂膝拗步) |
|  | Homework | 1. Reding materials:    - Observing your own bodily responses    - Body scan 2. Taiyi Mirror Heart Chuan practice 3. Mindfulness practice: mindfulness activities in daily life | 1. 16-form Yang-style Tai Chi Chuan practice |

|  |  | MTCC program  (Beginner stage: Session 1 ‒ 9) | TCC program |
| --- | --- | --- | --- |
| Session 4 | Major contents | 1. Review & Discussion 2. Taiyi Mirror Heart Chuan: form 5−6    - Needle at Sea Bottom – Left (左海底针)    - Flash the Arm – Left (左闪通臂) 3. Mindfulness practice: mindfulness of pleasant events | 1. Review 2. 16-form Yang-style Tai Chi Chuan: form 5–6    - Step, Parry and Punch (进步搬拦捶)    - Apparent Close Up (如封似闭) |
|  | Homework | 1. Reding materials:    - Seizing the moment    - The story of Nick Vujicic 2. Taiyi Mirror Heart Chuan practice 3. Mindfulness practice: pleasant events calendar | 1. 16-form Yang-style Tai Chi Chuan practice |
| Session 5 | Major contents | 1. Review & Discussion 2. Taiyi Mirror Heart Chuan: form 7−9    - Grasp the Bird’s Tail – Right (右揽雀尾)    - Needle at Sea Bottom – Right (右海底针)    - Flash the Arm – Right (右闪通臂) 3. Mindfulness practice: loving kindness meditation | 1. Review 2. 16-form Yang-style Tai Chi Chuan: form 7–8    - Single Whip (单鞭)    - Play Pipa (手挥琵琶) |
|  | Homework | 1. Reding materials:    - Caring for yourself 2. Taiyi Mirror Heart Chuan practice 3. Mindfulness practice: loving kindness meditation | 1. 16-form Yang-style Tai Chi Chuan practice |
| Session 6 | Major contents | 1. Review & Discussion 2. Taiyi Mirror Heart Chuan: dorm 10−11    - Repulse Monkey on Both Side (左右倒卷肱)    - Single Pushing Hand on Flat Round (平圆单推手) 3. Mindfulness practice: breathing space | 1. Review 2. 16-form Yang-style Tai Chi Chuan: form 9–10    - Repulse Monkey on Both Side (左右倒卷肱)    - Jade Lady Weaves Shuttles (左右穿梭) |
|  | Homework | 1. Reding materials:    - Breathing space 2. Taiyi Mirror Heart Chuan practice 3. Mindfulness practice: unpleasant events calendar | 1. 16-form Yang-style Tai Chi Chuan practice |

|  |  | MTCC program  (Beginner stage: Session 1 ‒ 9) | TCC program |
| --- | --- | --- | --- |
| Session 7 | Major contents | 1. Review & Discussion 2. Taiyi Mirror Heart Chuan: Form 12−13    - Single Pushing Hand of Fold (折叠单推手)    - Withdrawing the Step and Rollback (大捋) 3. Mindfulness practice: living with stress | 1. Review 2. 16-form Yang-style Tai Chi Chuan: form 11–13    - Needle at Sea Bottom (海底针)    - Flash the Arm (闪通臂)    - Wave Hands Like Clouds (云手) |
|  | Homework | 1. Reding materials:    - Introduction to Tai Chi Push Hands    - The open mind 2. Taiyi Mirror Heart Chuan practice 3. Mindfulness practice: breathing space | 1. 16-form Yang-style Tai Chi Chuan practice |
| Session 8 | Major contents | 1. Review & Discussion 2. Taiyi Mirror Heart Chuan: Form 14−16    - Prostrating and Looking Upward (昂首仰天式)    - Swinging the Tail (掉尾式)    - Closing Form (收势) 3. Mindfulness practice: cultivate gratitude heart | 1. Review 2. 16-form Yang-style Tai Chi Chuan: form 14–16    - Grasp the Bird's Tail on Both Side (左右揽雀尾)    - Cross Hands (十字手)    - Closing Form (收势) |
|  | Homework | 1. Reding materials:    - The life wisdom in Tai Chi Chuan    - A grateful heart 2. Taiyi Mirror Heart Chuan practice 3. Mindfulness practice: loving kindness meditation; daily recording what you are grateful for | 1. 16-form Yang-style Tai Chi Chuan practice |
| Session 9 | Major contents | 1. Introduction to mindfulness day 2. Review, share, and discuss personal experiences in the program 3. Closing mindfulness practice: Brief Loving Kindness Meditation | 1. Review: 16-form Yang-style Tai Chi Chuan 2. Discussion and summary |
|  | Homework | 1. Reding materials:    - The mindfulness journey: bring mindfulness into your life 2. Taiyi Mirror Heart Chuan practice 3. Mindfulness practice: experience and record mindfulness day | 1. 16-form Yang-style Tai Chi Chuan practice |

|  |  | MTCC program  (Consolidation stage: Session 10 ‒ 24) | TCC program |
| --- | --- | --- | --- |
| Session 10 | Major contents | 1. Discussion: sharing your mindfulness day 2. Introduction    - 24-form Yang-style Tai Chi Chuan    - 9 attitudes of mindfulness – Beginner’s Mind 3. Mindfulness practice: mindful breathing | 1. Introduction: 24-form Yang-style Tai Chi Chuan |
|  | Homework | 1. Mindfulness journal prompt: Notice the tastes, colors, smells, and textures of the food when eating. 2. Mindfulness practice: mindful breathing | / |
| Session 11 | Major contents | 1. Discussion: mindfulness journal 2. 24-form Yang-style Tai Chi Chuan: form 0‒2    - Preparation: take three deep breaths    - Commencing form (起势)    - Part the Wild Horse's Mane on Both Side (左右野马分鬃) 3. Mindfulness practice: body scan | 1. 24-form Yang-style Tai Chi Chuan: form 0‒2    - Preparation: take three deep breaths    - Commencing form (起势)    - Part the Wild Horse's Mane on Both Side (左右野马分鬃) |
|  | Homework | 1. 24-form Yang-style Tai Chi Chuan practice 2. Mindfulness journal prompt: Pause, and notice the beauty around you. 3. Mindfulness practice: body scan | 1. 24-form Yang-style Tai Chi Chuan practice |
| Session 12 | Major content | 1. Review & Discussion 2. 24-form Yang-style Tai Chi Chuan: form 3−4    - White Crane Spreads its Wings (白鹤亮翅)    - Brush Knee and Twist Step on Both Side (左右搂膝拗步) 3. Mindfulness practice: breathing space | 1. Review 2. 24-form Yang-style Tai Chi Chuan: form 3−4    - White Crane Spreads its Wings (白鹤亮翅)    - Brush Knee and Twist Step on Both Side (左右搂膝拗步) |
|  | Homework | 1. 24-form Yang-style Tai Chi Chuan practice 2. Mindfulness journal prompt: Pause, and notice the smells around you. 3. Mindfulness practice: experience and record mindfulness day | 1. 24-form Yang-style Tai Chi Chuan practice |

|  |  | MTCC program  (Consolidation stage: Session 10 ‒ 24) | TCC program |
| --- | --- | --- | --- |
| Session 13 | Major content | 1. Review & Discussion 2. 24-form Yang-style Tai Chi Chuan: form 5−6    - Play Pipa (手挥琵琶)    - Repulse Monkey (倒卷肱) 3. Mindfulness practice: loving kindness meditation | 1. Review 2. 24-form Yang-style Tai Chi Chuan: form 5−6    - Play Pipa (手挥琵琶)    - Repulse Monkey (倒卷肱) |
|  | Homework | 1. 24-form Yang-style Tai Chi Chuan practice 2. Mindfulness journal prompt: Pause, and notice the sounds around you. 3. Mindfulness practice: loving kindness meditation | 1. 24-form Yang-style Tai Chi Chuan practice |
| Session 14 | Major content | 1. Review & Discussion 2. 24-form Yang-style Tai Chi Chuan: form 7−8    - Grasp the Bird's Tail – Left (左揽雀尾)    - Grasp the Bird's Tail – Right (右揽雀尾) 3. Mindfulness practice: notice the pleasant moments | 1. Review 2. 24-form Yang-style Tai Chi Chuan: form 7−8    - Grasp the Bird's Tail – Left (左揽雀尾)    - Grasp the Bird's Tail – Right (右揽雀尾) |
|  | Homework | 1. 24-form Yang-style Tai Chi Chuan practice 2. Mindfulness journal prompt: Pause, and put your mind to one thing. 3. Mindfulness practice: experience and record mindfulness day | 1. 24-form Yang-style Tai Chi Chuan practice |
| Session 15 | Major content | 1. Review & Discussion 2. 24-form Yang-style Tai Chi Chuan: form 9−10    - Single Whip (单鞭)    - Wave Hands Like Clouds (云手) 3. Introduction: 9 attitudes of mindfulness − Nonjudgment 4. Mindfulness practice: body scan | 1. Review 2. 24-form Yang-style Tai Chi Chuan: form 9−10    - Single Whip (单鞭)    - Wave Hands Like Clouds (云手) |
|  | Homework | 1. 24-form Yang-style Tai Chi Chuan practice 2. Mindfulness journal prompt: Pause, and pay attention to the things in your life that are about to be evaluated or perceived to be evaluated. Try not to evaluate, and continue to write about your feelings with original insight, experience, acceptance, and understanding. 3. Mindfulness practice: mindful breathing; body scan | 1. 24-form Yang-style Tai Chi Chuan practice |

|  |  | MTCC program  (Consolidation stage: Session 10 ‒ 24) | TCC program |
| --- | --- | --- | --- |
| Session 16 | Major content | 1. Review & Discussion 2. 24-form Yang-style Tai Chi Chuan: form 11−12    - Single Whip (单鞭)    - High Pat on Horse (高探马) 3. Introduction: 9 attitudes of mindfulness – Acceptance 4. Mindfulness practice: body scan | 1. Review & Discussion 2. 24-form Yang-style Tai Chi Chuan: form 11−12    - Single Whip (单鞭)    - High Pat on Horse (高探马) |
|  | Homework | 1. 24-form Yang-style Tai Chi Chuan practice 2. Mindfulness journal prompt: Pause, try to accept and understand the things or moments in your life that you are resisting or preparing to resist (specifically, things or moments that are objective and cannot be changed for the time being), and write about your feelings. 3. Mindfulness practice: experience and record mindfulness day | 1. 24-form Yang-style Tai Chi Chuan practice |
| Session 17 | Major content | 1. Review & Discussion 2. 24-form Yang-style Tai Chi Chuan: form 13−14    - Kick with Right Heel (右蹬脚)    - Strike Opponent's Ears with Both Fists (双峰贯耳) 3. Introduction: 9 attitudes of mindfulness – Patience 4. Mindfulness practice: loving kindness meditation | 1. Review & Discussion 2. 24-form Yang-style Tai Chi Chuan: form 13−14    - Kick with Right Heel (右蹬脚)    - Strike Opponent's Ears with Both Fists (双峰贯耳) |
|  | Homework | 1. 24-form Yang-style Tai Chi Chuan practice 2. Mindfulness journal prompt: Pause, and try to be aware and accepting of the impatient moment when practicing Tai Chi Chuan, or the moment in daily life. Write about your feelings. 3. Mindfulness practice: loving kindness meditation | 1. 24-form Yang-style Tai Chi Chuan practice |
| Session 18 | Major content | 1. Discussion 2. Midterm review: 24-form Yang-style Tai Chi Chuan form 1−14 3. Introduction: 9 attitudes of mindfulness – Trust 4. Mindfulness practice: body scan | 1. Midterm review: 24-form Yang-style Tai Chi Chuan form 1−14 2. Discussion and summary |
|  | Homework | 1. 24-form Yang-style Tai Chi Chuan practice 2. Mindfulness journal prompt: Record a moment of trust in yourself or others. 3. Mindfulness practice: experience and record mindfulness day | 1. 24-form Yang-style Tai Chi Chuan practice |

|  |  | MTCC program  (Consolidation stage: Session 10 ‒ 24) | TCC program |
| --- | --- | --- | --- |
| Session 19 | Major content | 1. Review & Discussion 2. 24-form Yang-style Tai Chi Chuan: form 15−16    - Turn and Kick with Left Heel (转身左蹬脚)    - Snake Creeps Down – Left (左下独立式) 3. Introduction: 9 attitudes of mindfulness – Letting Go 4. Mindfulness practice: loving kindness meditation | 1. Review 2. 24-form Yang-style Tai Chi Chuan: form 15−16    - Turn and Kick with Left Heel (转身左蹬脚)    - Snake Creeps Down – Left (左下独立式) |
|  | Homework | 1. 24-form Yang-style Tai Chi Chuan practice 2. Mindfulness journal prompt: Write down one or two recent thoughts or moments that you can't take it away, try to let it go. 3. Mindfulness practice: experience and record mindfulness day | 1. 24-form Yang-style Tai Chi Chuan practice |
| Session 20 | Major content | 1. Review & Discussion 2. 24-form Yang-style Tai Chi Chuan: form 17−18    - Snake Creeps Down – Right (右下独立式)    - Jade Lady Weaves Shuttles (左右穿梭) 3. Introduction: 9 attitudes of mindfulness – Non-Striving 4. Mindfulness practice: loving kindness meditation; breathing space | 1. Review 2. 24-form Yang-style Tai Chi Chuan: form 17−18    - Snake Creeps Down – Right (右下独立式)    - Jade Lady Weaves Shuttles (左右穿梭) |
|  | Homework | 1. 24-form Yang-style Tai Chi Chuan practice 2. Mindfulness journal prompt: Write about a recent event or moment that you would have been striving or forcing, but are not striving now. How do you feel? 3. Mindfulness practice: loving kindness meditation; breathing space | 1. 24-form Yang-style Tai Chi Chuan practice |
| Session 21 | Major content | 1. Review & Discussion 2. 24-form Yang-style Tai Chi Chuan: form 19−20    - Needle at Sea Bottom (海底针)    - Flash the Arm (闪通臂) 3. Mindfulness practice: notice the unpleasant moments and try to accept; breathing space | 1. Review 2. 24-form Yang-style Tai Chi Chuan: form 19−20    - Needle at Sea Bottom (海底针)    - Flash the Arm (闪通臂) |
|  | Homework | 1. 24-form Yang-style Tai Chi Chuan practice 2. Mindfulness journal prompt: This week, what is the most impressive or interesting knowledge do you think when you attended a class that usually bored you? 3. Mindfulness practice: experience and record mindfulness day | 1. 24-form Yang-style Tai Chi Chuan practice |
|  |  | **MTCC program**  **(Consolidation stage: Session 10 ‒ 24)** | **TCC program** |
| Session 22 | Major content | 1. Review & Discussion 2. 24-form Yang-style Tai Chi Chuan: form 21−22    - Turn, Deflect Downward, Parry and Punch (转身搬拦捶)    - Apparent Close up (如封似闭) 3. Introduction: 9 attitudes of mindfulness – Gratitude 4. Mindfulness practice: loving kindness meditation | 1. Review 2. 24-form Yang-style Tai Chi Chuan: form 21−22    - Turn, Deflect Downward, Parry and Punch (转身搬拦捶)    - Apparent Close up (如封似闭) |
|  | Homework | 1. 24-form Yang-style Tai Chi Chuan practice 2. Mindfulness journal prompt: List 1–3 parts of your body that you are most satisfied with. 3. Mindfulness practice: loving kindness meditation | 1. 24-form Yang-style Tai Chi Chuan practice |
| Session 23 | Major content | 1. Review & Discussion 2. 24-form Yang-style Tai Chi Chuan: form 23−24    - Cross Hands (十字手)    - Closing Form (收势) 3. Introduction: 9 attitudes of mindfulness – Generosity 4. Mindfulness practice: breathing space | 1. Review 2. 24-form Yang-style Tai Chi Chuan: form 23−24    - Cross Hands (十字手)    - Closing Form (收势) |
|  | Homework | 1. 24-form Yang-style Tai Chi Chuan practice 2. Mindfulness journal prompt: Which good habits, although small, have benefited you all the time? 3. Mindfulness practice: experience and record mindfulness day | 1. 24-form Yang-style Tai Chi Chuan practice |
| Session 24 | Major content | 1. Review & Discussion 2. 24-form Yang-style Tai Chi Chuan practice 3. Review, share and discuss personal experiences from the course | 1. 24-form Yang-style Tai Chi Chuan practice 2. Review, share and discuss personal experiences from the course |

Note: All mindfulness practices in this intervention program are selected from 8-week Mindfulness-Based Stress Reduction (MBSR) program (Kabat-Zinn, 1990).

***“Mindful sharing” in Review & Discussion***

Script: Today, we’ll focus on your experiences during this week’s practice—there are no “right” answers, just honest reflections. Let’s start with 30 seconds of silent grounding. When ready, we’ll take turns; please speak from your body and feelings, and listeners, let’s practice non-judgmental presence.
