# Supplementary material for: Improving exercise motivation and physical fitness in college students through a long-term mindfulness-enhanced Tai Chi Chuan program: a randomized controlled trial
Source: PeerJ. 2026 Jan 9;14:e20602. doi: 10.7717/peerj.20602 (PMC12794632; doi:10.7717/peerj.20602)
Supplement: Supplemental Information 3 [file peerj-14-20602-s003.pdf]

临床试验注册相关信息汇总

Title: The Effects of Mindfulness-incorporated Sports on the Physical and Mental Health of College Students

Registration No. ChiCTR2200058449

审核状态：<br>Project audit state：	该项目已经通过审核，不能再修改项目信息。<br>This trial has been verified,you can't edit it any more.<br>返回Back
注册号：<br>Registration number：	ChiCTR2200058449
最近更新日期：<br>Date of Last Refreshed on：	2022/4/9 20:38:37
注册号状态：	预注册
Registration Status：	1008001 Prospective registration
注册题目：	融入正念的体育运动对大学生身心健康的影响
Public title：	The Effects of Mindfulness-incorporated Sports on the Physical and Mental Health of College Students
研究课题的正式科学名称：	融入正念的体育运动对大学生身心健康的影响
Scientific title：	The Effects of Mindfulness-incorporated Sports on the Physical and Mental Health of College Students
研究课题代号(代码)：<br>Study subject ID：	
在其它机构的注册号：<br>Secondary ID：	

申请注册联系人：	屈萍	研究负责人：	屈萍
Applicant：	Qu Ping	Study leader：	Qu Ping
申请注册联系人电话：<br>Applicant telephone：	13560477720	研究负责人电话：<br>Study leader's telephone：	13560477720
申请注册联系人传真 ：<br>Applicant Fax：		研究负责人传真：<br>Study leader's fax：	
申请注册联系人电子邮件：<br>Applicant E-mail：	87933887@qq.com	研究负责人电子邮件：<br>Study leader's E-mail：	87933887@qq.com
申请单位网址(自愿提供)：<br>Applicant website(voluntary supply)：		研究负责人网址(自愿提供)：<br>Study leader's website(voluntary supply)：	
申请注册联系人通讯地址：	广州市海珠区新港西路135号中山大学	研究负责人通讯地址：	广州市海珠区新港西路135号中山大学
Applicant address：	Sun Yat-sen University, No. 135,West Xingang Road, Guangzhou	Study leader's address：	Sun Yat-sen University, No. 135, West Xingang Road, Guangzhou
申请注册联系人邮政编码：<br>Applicant postcode：	510275	研究负责人邮政编码：<br>Study leader's postcode：	510275
申请人所在单位：	广州市海珠区新港西路135号中山大学		
Applicant's institution：	Sun Yat-sen University, No. 135, West Xingang Road, Guangzhou		

是否获伦理委员会批准：	是		
Approved by ethic committee：	Yes		
伦理委员会批件文号：<br>Approved No. of ethic committee：	2021-1105-0213	伦理委员会批件附件：<br>Approved file of Ethical Committee：	查看附件View
批准本研究的伦理委员会名称：	中山大学心理学系保护人类被试伦理审查委员会		
Name of the ethic committee：	Ethics Review Committee for the Protection of Human Subjects, Department of Psychology, Sun Yat -sen University		
伦理委员会批准日期：<br>Date of approved by ethic committee：	2021/12/31		
伦理委员会联系人：	屈萍		
Contact Name of the ethic committee：	Ping Qu		
伦理委员会联系地址：	广州市海珠区新港西路135号中山大学		
Contact Address of the ethic committee：	Sun Yat-sen University, No. 135, West Xingang Road, Guangzhou		
伦理委员会联系人电话：<br>Contact phone of the ethic committee：	13560477720	伦理委员会联系人邮箱：<br>Contact email of the ethic committee：	87933887@qq.com

研究实施负责（组长）单位：中山大学体育部

Primary sponsor：Department of Physical Education, Sun Yat-sen University

研究实施负责（组长）单位地址：广州市海珠区新港西路135号

Primary sponsor's address：West Xingang Road, Guangzhou

试验主办单位(项目批准或申办者)：
Secondary sponsor：

国家：	中国	省(直辖市)：	广东	市(区县)：	广州
Country：	China	Province：	Guang Dong	City：	Guangzhou
单位(医院)：	中山大学	具体地址：	广州市海珠区新港西路135号		
Institution hospital：	Sun Yat-sen University	Address：	Sun Yat-sen University, No. 135, West Xingang Road, Guangzhou		

经费或物资来源：中山大学体育部

Source(s) of funding：Department of Physical Education, Sun Yat-sen University

研究疾病：心理健康问题、体质健康水平下降

Target disease：Mental Health Problems, Physical Health Worsening

研究疾病代码：

Target disease code：

研究类型：干预性研究

Study type：Interventional study

研究所处阶段：I期临床试验

Study phase：1

研究目的：大学生的身心健康关乎国家未来，正念干预体育运动课程在大学生群体的积极作用已经得到大量研究支持。本研究结合心理学和体育学的理论及实证研究，开发了融合了正念要素的体育教育课程（如:正念太极拳套路、正念健身等）及干预方法，以期同时促进个体的身体健康和心理健康。同时，本研究也尝试把正念与更多体育运动项目相结合，进一步考察融入正念的体育运动对大学生身心健康的促进作用。本研究计划在考察融入正念的体育运动干预项目在大学生群体中的可行性和有效性，并进一步探讨其作用机制。

Objectives of Study：The physical and mental health of college students is related to the future of the country, and the positive role of mindfulness-based intervention in physical education in college students has been supported by a lot of research. Combining theoretical and empirical research in psychology and physical education, this study develops physical education courses (such as mindfulness Taijiquan routines, mindfulness fitness, etc.) and intervention methods that incorporate mindfulness elements, with a view to promoting individual physical and mental health at the same time. . At the same time, this study also tried to combine mindfulness with more sports to further investigate the effect of mindfulness-incorporated sports on the physical and mental health of college students. This study plans to investigate the feasibility and effectiveness of mindfulness-incorporated sports interventions in college students, and to further explore its mechanism of action.elements, with a view to promoting individual physical and mental health at the same time. . At the same time, this study also tried to combine mindfulness with more sports to further investigate the effect of mindfulness-incorporated sports on the physical and mental health of college students. This study plans to investigate the feasibility and effectiveness of mindfulness-incorporated sports interventions in college students, and to further explore its mechanism of action.

研究设计：随机平行对照

Study design：Parallel

纳入标准：1、身心健康状况稳定，无罹患高血压、冠心病、糖尿病等高危疾病或精神心理疾病，无不良生活习惯（吸烟、酗酒等）；2、大一、大二在校学生；3、未接受过系统正念训练等。

Inclusion criteria 1. Stable physical and mental health, no high-risk diseases such as hypertension, coronary heart disease, diabetes, or mental and psychological diseases, and no bad living habits (smoking, alcoholism, etc.); 2. Freshman and sophomore students; 3. Have not received systematic mindfulness training, etc.

排除标准：1、罹患高血压、冠心病、糖尿病等高危疾病或精神心理疾病、身体残疾或运动系统疾病的人群；2、长期进行系统的太极拳、抗阻训练或其他相关训练等。

Exclusion criteria：1. People suffering from high-risk diseases such as hypertension, coronary heart disease, diabetes, or mental and psychological diseases, physical disabilities or motor system diseases; 2. Conduct systematic Tai Chi, resistance training or other related training for a long time.

研究实施时间：
Study execute time：从From2021/10/08至To 2023/02/15

干预措施：
Interventions：

组别：	正念太极拳组	样本量：	50
Group：	Mindfulness Tai Chi Chuan Group	Sample size：	
干预措施：	进行《太易镜心拳》正念太极课程教学	干预措施代码：	
Intervention：	正念太极拳组	Intervention code：	
组别：	传统太极拳组	样本量：	50
Group：	Traditional Tai Chi Chuan Group	Sample size：	
干预措施：	进行杨氏十六式太极拳入门基础套路教学	干预措施代码：	
Intervention：	传统太极拳组	Intervention code：	
组别：	正念力量（抗阻）训练组	样本量：	50
Group：	Mindfulness Fitness Group	Sample size：	
干预措施：	融入正念的抗阻训练教学	干预措施代码：	
Intervention：	正念力量（抗阻）训练组	Intervention code：	
组别：	传统力量（抗阻）训练组	样本量：	50
Group：	Traditional Fitness Group	Sample size：	
干预措施：	按照中山大学健身课标准教案进行教学	干预措施代码：	
Intervention：	传统力量（抗阻）训练组	Intervention code：	
组别：	融入正念的其它体育运动组	样本量：	150
Group：	Other sports groups that incorporate mindfulness	Sample size：	
干预措施：	融入正念的相关体育运动项目教学	干预措施代码：	
Intervention：	融入正念的其它体育运动组	Intervention code：	
组别：	传统其它体育运动组	样本量：	150
Group：	Traditional other sports group	Sample size：	
干预措施：	按照相关课程标准教案进行教学	干预措施代码：	
Intervention：	传统其它体育运动组	Intervention code：	

研究实施地点：
Countries of recruitment and research settings：

国家：	中国	省(直辖市)：	广东	市(区县)：	广州
Country：	China	Province：	Guang Dong	City：	Guangzhou
单位(医院)：	中山大学	单位级别：			
Institution hospital：	Sun Yat-sen University	Level of the institution：			

测量指标：
Outcomes：

指标中文名：	运动动机		
Outcome：	Sports Motivation		
测量时间点：	2022.04、2023.01	测量方法：	使用《锻炼动机量表（BREQ-2）》进行问卷调查
Measure time point of outcome：	2022.04，2023.01	Measure method：	Questionnaire survey using the Exercise Motivation Scale (BREQ-2)

指标中文名：	学习效率		
Outcome：	Learning Efficiency		
测量时间点：	2022.04、2023.01	测量方法：	使用《学业自我效能感量表》进行问卷调查
Measure time point of outcome：	2022.04，2023.01	Measure method：	Questionnaire survey using the Academic Self-Efficacy Scale

指标中文名：	自尊水平		
Outcome：	Self-esteem Level		
测量时间点：	2022.04、2023.01	测量方法：	使用《自尊水平量表》进行问卷调查
Measure time point of outcome：	2022.04，2023.01	Measure method：	Questionnaire survey using the Self-Esteem Level Scale

指标中文名：	生活质量		
Outcome：	Living Standard		
测量时间点：	2022.04、2023.01	测量方法：	使用《健康促进生活方式量表》进行问卷调查
Measure time point of outcome：	2022.04，2023.01	Measure method：	Questionnaire survey using the Health Promotion Lifestyle Scale

指标中文名：	身体素质		
Outcome：	Physical Fitness		
测量时间点：	2022.04、2023.01	测量方法：	大学生体质健康测试
Measure time point of outcome：	2022.04，2023.01	Measure method：	College Students' Physical Health Evaluation

指标中文名：	幸福感		
Outcome：	Happiness		
测量时间点：	2022.04、2023.01	测量方法：	使用《主观幸福感量表》进行问卷调查
Measure time point of outcome：	2022.04，2023.01	Measure method：	Questionnaire survey using the Subjective Well-Being Scale

指标中文名：	身体形态		
Outcome：	Body Shape		
测量时间点：	2022.04、2023.01	测量方法：	大学生体质健康测试
Measure time point of outcome：	2022.04，2023.01	Measure method：	College Students' Physical Health Evaluation

指标中文名：	正念水平		
Outcome：	Mindfulness Standard		
测量时间点：	2022.04、2023.01	测量方法：	使用《正念五因素量表（FFMQ)》进行问卷调查
Measure time point of outcome：	2022.04，2023.01	Measure method：	Questionnaire survey using the Five-Factor Mindfulness Scale (FFMQ)

指标中文名：	健康行为		
Outcome：	Healthy Behavior		
测量时间点：	2022.04、2023.01	测量方法：	使用《健康促进生活方式量表（HPLP-II）》进行问卷调查
Measure time point of outcome：	2022.04，2023.01	Measure method：	Questionnaire survey using the Health-Promoting Lifestyle Scale (HPLP-II)

采集人体标本：<br>Collecting sample(s) from participants：	标本中文名：受试者 ；组织：<br>Sample Name： ；Tissue：<br>人体标本去向：其它 ；说明<br>Fate of sample：Others ；Note：
征募研究对象情况：<br>Recruiting status：	结束<br>Completed
年龄范围：<br>Participant age：	最小 Min age 18 岁 years<br>最大 Max age 22 岁 years
性别：	男女均可
Gender：	Both
随机方法（请说明由何人用什么方法产生随机序列）：	研究工作人员根据受试者数量，本研究采用2*2随机对照的实验方法，由SAS统计软件用分层随机化法产生随机数字表。
Randomization Procedure (please state who generates the random number sequence and by what method)：	According to the number of subjects, the research staff adopts the experimental method of 2*2 random control, and the random number table is generated by the stratified randomization method by SAS statistical software.
盲法：	
Blinding：	
是否公开试验完成后的统计结果：<br>Calculated Results ater the Study Completed public access：	不公开/Private 变更change
上传的试验完成后的统计结果：<br>Statistical results after completion of the test file upload：	重传Upload： 选择文件 未选择任何文件 *
UTN(全球唯一识别码)：	
是否共享原始数据：<br>IPD sharing	否No
共享原始数据的方式（说明：请填入公开原始数据日期和方式，如采用网络平台，需填该网络平台名称和网址）：	心理学和体育学学术杂志稿件、中山大学本科生和研究生毕业论文、心理学和体育学专业会议
The way of sharing IPD"(include metadata and protocol, If use web-based public database, please provide the url)：	Manuscripts for academic journals of psychology and physical education, undergraduate and postgraduate graduation thesis of Sun Yat-sen University, professional conferences on psychology and physical
数据采集和管理（说明：数据采集和管理由两部分组成，一为病例记录表(Case Record Form, CRF)，二为电子采集和管理系统(Electronic Data Capture, EDC)，如ResMan即为一种基于互联网的EDC。：	1、心理健康测量问卷由研究人员发放、回收、录入和分析；结果报告时不包括任何被试的个人信息； 2、执行功能测试由心理系研究助理进行测试、分析；结果报告时不包括任何被试的个人信息； 3、体适能测试由体育部研究助理对被试进行逐一测试和记录测试成绩； 4、结果报告时不包括任何被试的个人信息； 5、心理健康问卷和执行功能数据，以及体质评估、体成分、体适能测试成绩将由研究人员保管，数据和信息储存在电脑里，并设置密码。
Data collection and Management (A standard data collection and management system include a CRF and an electronic data capture：	1. The mental health measurement questionnaire is issued, recovered, entered and analyzed by the researchers; the results report does not include any personal information of the subjects; 2. The executive function test is tested and analyzed by the research assistant of the Department of Psychology; the results report does not include any personal information of the subjects; 3. In the physical fitness test, the research assistant of the Ministry of Sports will test the subjects one by one and record the test results; 4. The result report does not include any personal information of the subjects; 5. The mental health questionnaire and executive function data, as well as physical assessment, body composition, and physical fitness test scores will be kept by the researchers, and the data and information will be stored in the computer, and a password will be set.
数据与安全监察委员会：<br>Data and Safety Monitoring Committee：	暂未确定/Not yet
研究计划书或研究结果报告发表信息（杂志名称、期、卷、页，时间；或网址）：	
Publication information of the protocol/research results report (name of the journal, volume, issue, pages, time; or website)：	修改/update
注册人：<br>Name of Registration：	2022/04/09
项目来源：<br>Project Origin：	本站

返回Back
