# Supplementary material for: Improving exercise motivation and physical fitness in college students through a long-term mindfulness-enhanced Tai Chi Chuan program: a randomized controlled trial
Source: PeerJ. 2026 Jan 9;14:e20602. doi: 10.7717/peerj.20602 (PMC12794632; doi:10.7717/peerj.20602)
Supplement: Supplemental Information 5 [file peerj-14-20602-s005.docx]

**Categorical Data Codebook​**

1. Document Description​

This codebook is designed to clearly define the categorical data recorded numerically in the original research dataset. It specifies the exact categorical factor corresponding to each numerical code, ensuring the accuracy and reproducibility of data interpretation. This document only covers the two categorical variables that were numerically encoded in the dataset, with details on variable names, numerical codes, and corresponding factors provided below.​

2. Variable Code Mapping Table

| Variable Name | Numerical Code | Corresponding Factor |
| --- | --- | --- |
| gender | 1 | Male |
|  | 2 | Female |
| group | 0 | TCC (Tai Chi Chuan) group |
|  | 1 | MTCC (Mindfulness-enhanced Tai Chi Chuan) group |
